# Early and Long-Term Outcomes of Carotid Stenting and Carotid Endarterectomy in Women

**DOI:** 10.3389/fsurg.2021.646204

**Published:** 2021-03-08

**Authors:** Edoardo Pasqui, Gianmarco de Donato, Giuseppe Alba, Brenda Brancaccio, Claudia Panzano, Alessandro Cappelli, Carlo Setacci, Giancarlo Palasciano

**Affiliations:** Vascular Surgery Unit, Department of Medicine, Surgery, and Neuroscience, University of Siena, Siena, Italy

**Keywords:** carotid disease, carotid stenting, carotid endarterectomy, women, stroke

## Abstract

**Background:** The role of carotid revascularization in women remains intensely debated because of the lower benefit and higher perioperative risks concerning the male counterpart. Carotid artery endarterectomy (CEA) and stenting (CAS) represent the two most valuable stroke prevention techniques due to large vessel disease. This study investigates the early and late outcomes in female sex in a real-world everyday clinical practice.

**Methods:** Data were retrospectively analyzed from a single-center database prospectively compiled. A total of 234 procedures, both symptomatic and asymptomatic, were identified (98 CEAs and 136 CASs). Perioperative risks of death, cerebral ischemic events, and local complications were analyzed and compared between the two groups. Long-term outcomes were evaluated in overall survival, freedom from ipsilateral stroke/transient ischemic attack, and freedom from restenosis (>50%) and reintervention.

**Results:** Women who underwent CAS and CEA did not differ in perioperative ischemic cerebral events (2.2 vs. 0%, *p* = 0.26) and death (0.8 vs. 0%, *p* = 1). Other perioperative and 30-day outcomes were similarly distributed within the two groups. Kaplan–Meier curves between CAS and CEA groups highlighted no statistical differences at 6 years in overall survival (77.4 vs. 77.1%, *p* = 0.47) of ipsilateral stroke/transient ischemic attack (94.1 vs. 92.9%, *p* = 0.9). Conversely, significant differences were showed in 6 years freedom from restenosis (93.1 vs. 83.3%, *p* = 0.03) and reinterventions (97.7 vs. 87.8%, *p* = 0.015).

**Conclusion:** Our results revealed that both CEA and CAS have acceptable perioperative risk in women. Long-term outcomes highlighted favorable indications for both procedures, especially for CAS, which seemed to be an excellent alternative to CEA in female patients when performed by well-trained operators.

## Introduction

Stroke remains one of the top causes of death and disability worldwide, imposing a substantial socioeconomic burden (1). It represents the fourth and fifth leading cause of death in women and men, respectively.

Extracranial carotid artery disease represents a substantial cause of stroke: ~7–12% of all strokes and 9 to 15% of all ischemic strokes (2).

The choice between carotid artery endarterectomy (CEA) and carotid artery stenting (CAS) remains debated, especially in patients' specific cohorts as female patients (lethality, disability, and reduced quality of life) (3).

Besides, due to the increased life expectancy of women and the higher lifetime risk of stroke (4), the rising relevance of the impact of stroke on female patients has emerged, also trying to determine specific features of carotid atherosclerotic plaques [more “stable” phenotype compared with men's plaque, (5) minor mean area of stenosis (6), different plaque location (7), and higher age-related arterial stiffness (8)] to understand what the best treatment is.

Although benefits of preventive treatments may be uncertain, female sex has been enlisted as a potential factor affecting poor perioperative outcomes after CEA (9–11) and increasing the risk of periprocedural events related to the aortic arch and carotid artery catheter manipulation.

The present study aimed to analyze a high volume of single-center experience in perioperative outcomes and the long-term effectiveness of carotid revascularization (CAS and CEA) in female patients.

## Methods

### Patients Population

All consecutive female patients who underwent primary extracranial carotid revascularization from January 2013 to December 2019 were included in the study cohort. Indication for revascularization followed the North American Symptomatic Carotid Endarterectomy Trial criteria (12), the degree of stenosis and related symptoms: symptomatic stenosis of the internal carotid artery >50% and asymptomatic stenosis >80%.

Patients with amaurosis fugax, hemispheric transient ischemic attack (TIA), or ipsilateral ischemic stroke with and without a major disability were considered symptomatic if they occurred 6 months before the intervention.

Patients' demographics, intraoperative data, and postoperative outcomes were collected through hospital charts. Coronary artery disease, hypertension, diabetes mellitus, chronic obstructive pulmonary disease, renal disease (chronic renal insufficiency defined by serum creatinine >1.2 mg/dl), smoking history (any current or past regular use of tobacco), dyslipidemia, atrial fibrillation, and peripheral artery disease were taken in account as comorbidities.

Gynecological history was also explored: menopause age, number of pregnancies, and history of abortion were registered. The decision to include these data was related to the possible association of the number of pregnancies and pregnancy losses and subsequent risks of myocardial infarction, cerebral infarction, and renovascular hypertension, consistent with either shared etiology or the initiation of pathological leading to atherosclerotic disease (13, 14).

### Carotid Endarterectomy and Carotid Stenting Techniques

The decision to perform CEA or CAS was based on the patient's comprehensive evaluation: baseline patients' comorbidities, atherosclerotic plaque characterization, arch type, supra-aortic vessel morphology, neurological status, and surgeons' and patients' preferences.

At that time, in our institution, staff surgeons had extensive experience in both techniques (more than 500 cases per surgeon) with a documented low rate of perioperative stroke and death (15–17), using a standardized protocol.

CAS was performed using a transfemoral approach under local anesthesia. Cerebral hemispheric perfusion was continuously assessed using the near-infrared spectroscopy system when possible. Systemic heparinization was reached with intravenous heparin (100 UI/kg), near after 8 for introducer sheath positioning in the common femoral artery. All procedures were performed using cerebral protection devices and various stent models. The choice between different types of stents was based on anatomical considerations and plaque-type (18–20). Stent dimensions were chosen according to the preoperative routine duplex ultrasonography (DUS) examination. Percutaneous arterial access hemostasis was achieved *via* manual compression or closure devices.

CEA was usually performed under a loco-regional blockade. Intra-procedural near-infrared spectroscopy was used to evaluate cerebral perfusion. An intra-procedural shift from loco-regional to general anesthesia could happen due to intra-procedural complications or patient's noncompliance. *Ab initio* general anesthesia was chosen in response to the collegial multidisciplinary evaluation. Preoperative DUS mapping of carotid bifurcation and plaque extension was performed routinely to center the skin incision and limit the skin incision length (21). The arterial shunt was used selectively according to clamp intolerance or high-risk patients (e.g., contralateral internal carotid occlusion). Systemic heparinization was used at the same dosage as CAS and consecutively reversed after arterial de-clamping. Patch-plasty or eversion techniques were used; the decision was made intraoperatively according to anatomical/technical considerations and surgeon preference.

### Patients Preparation and Medical Management

The preoperative degree of stenosis was assessed using DUS by experienced operators. The CAS-patients' primary attention was focused on the femoral artery accesses, carotid tortuosity, plaque length, and pre-lesion and post-lesion artery diameters.

Plaques characteristics were assessed during DUS exams, and in the CEA group, morphological assessment was confirmed intraoperatively.

Angio-CT was performed in patients candidate to CEA (especially in case of DUS uncertainty) and in all CAS patients to evaluate aortic arch status.

CAS patients received dual antiplatelet therapy that consisted of acetylsalicylic acid (75 to 100 mg/die) and clopidogrel (75 mg/die) for at least 1 week before the intervention and 1 month postoperatively. In patients who underwent CEA, single antiplatelet therapy was sufficient, without interruption in the perioperative time.

### Follow-Up

The scheduled follow-up consisted of a 30-day postoperative clinical and DUS examination performed by an experienced vascular surgeon, repeated at 6 and 12 months and yearly after that. In symptomatic carotid artery disease, a neurological assessment was also made at a 1-month follow-up. Angio-CT was requested to evaluate future complications.

### Outcomes

The early primary endpoint was the combined risk of any stroke or death within the first 30 days after the intervention. Early secondary endpoints were any neurological event (major and minor stroke and TIA), myocardial infarction (MI), and a composite minor early adverse events endpoint. Respectively, in the CEA group, we included: surgical access complications, cranial/cervical nerve injuries, and cerebral reperfusion syndrome; in the CAS group, we included: percutaneous access complications, renal impairment, and cerebral reperfusion syndrome.

Renal function deterioration was defined as an elevation of serum creatinine concentration of >25% or >0.5 mg/dl (44 mmol/L) from baseline within 48 h. Cerebral reperfusion syndrome was defined as a condition characterized by ipsilateral headache, hypertension, seizures, and focal neurological deficits.

Late outcomes consisted of an ipsilateral neurological event, death, restenosis, and reintervention rates.

Neurological complications were classified as follows: TIA was defined as a brief episode of neurological dysfunction caused by focal brain or retinal ischemia, with clinical symptoms typically lasting <1 h, and without evidence of acute infarction; minor stroke was defined as a new neurological deficit that ultimately resolved in 30 days or increased the National Institutes of Health Stroke Scale score by three points compared with the pre-procedural evaluation; major stroke was defined as a new neurological deficit that persisted for >30 days and increased the National Institutes of Health Stroke Scale score by four points compared with the pre-procedural evaluation.

Restenosis was defined as a narrowing of the treated carotid artery ≥50%, highlighted by DUS or CTA, considering the revised velocity criteria for carotid stenting (22).

The Institutional Review Board approved the study protocol and informed consent, and all subjects gave informed consent. The ethical committee of the hospital was informed of the no-experimental design of the retrospective investigation and endorsed the study.

### Statistical Analysis

Categorical data were reported as numbers and percentages. Means (± standard deviation) and medians were used to analyze continuous variables. Student two-tailed *t*-test was used when applicable, and categorical variables were compared using Fisher's exact test. Statistical significance was considered for a *p*-value of < 0.05. Rates of freedom from overall death, reintervention, and cerebral ischemic events were estimated with the Kaplan–Meier method. A log-rank test was used to compare life table curves.

All statistical analyses were performed with GraphPad Prism 8.0 (GraphPad Software Inc., San Diego, CA, United States) and StatPlus Build 7.1.1 (AnalysisSoft Inc. Walnut, CA, United States).

## Results

During the study period, a total of 234 procedures of carotid revascularizations were performed in women: 98 (41.9%) CEAs and 136 (58.1%) CASs, respectively.

Demographic baseline data are listed in Table 1.

**Table 1:** Baseline characteristics.

**Baseline Features**	**CEA group**	**CAS Group**	**p-Value**
Age (mean ± SD)	73.4 ± 8.2	76.4 ± 6.6	0.002
Hypertension (*N*, %)	49 (50%)	90 (66.2%)	0.01
Diabetes (*N*, %)	23 (23.4%)	42 (30.9%)	0.23
Chronic renal failure (*N*, %)	11 (11.2%)	19 (14%)	0.56
COPD (*N*, %)	6 (6.1%)	18 (13.2%)	0.08
Coronary disease (*N*, %)	13 (13.2%)	26 (19.1%)	0.29
Atrial fibrillation (*N*, %)	7 (7.1%)	12 (8.8%)	0.8
Smoke (*N*, %)	20 (20.4%)	50 (36.8%)	0.009
Former smoker	7 (7.1%)	26 (19.1%)	0.01
Dyslipidaemia (*N*, %)	46 (47%)	67 (49.3%)	0.8
PAD (*N*, %)	9 (9.2%)	15 (11%)	0.8
Menopause age (mean ± SD)	47.1 ± 4.3	49.5 ± 3.9	0.0001
Term pregnancies (*N*, %)			
Single	44 (44.9%)	43 (31.6%)	0.04
Multiple (>2)	47 (47.9%)	63 (46.3%)	0.9
History of abortion (*N*, %)	29 (29.6%)	53 (39%)	0.2

*SD, standard deviation; COPD, chronic obstructive pulmonary disease; PAD, peripheral artery disease*.

The vast majority of patients enrolled were asymptomatic in both groups (92.9 vs. 91.9% in CEA and CAS). Atherosclerotic plaque features are highlighted in Table 2. CEA patients were more likely to have a hypoechoic plaque (50 vs. 17.6% in CEA and CAS, respectively, *p* = 0.0001), and hyperechoic and isoechoic configurations were more represented in the CAS group.

**Table 2:** Carotid plaque features and neurological history.

	**CEA Group**	**CAS Group**	***p*-value**
Right ICA (*N*, %)	48 (50.5%)	76 (55.9%)	0.35
Average of stenosis (mean ± SD)	82.7 ± 5.8	82.5 ± 6.0	0.8
Average Max PSV (cm/s)	285 ± 56.2	264.4 ± 50.5	0.003
Average Max EDV (cm/s)	47.3 ± 13.5	67.0 ± 23.4	0.0001
Irregular Plaque profile	51 (52%)	66 (48.5%)	0.7
Echogenicity			
Mostly Ipoechoic	49 (50%)	24 (17.6%)	0.0001
Mostly Iperechoic	31 (31.6%)	62 (45.6%)	0.04
Mostly Isoechoic	18 (18.4%)	50 (36.8%)	0.002
Ulcerated plaque	25 (25.5%)	17 (12.5%)	0.01
Contralateral ICA occlusion	5 (5.1%)	12 (8.8%)	0.3
Symptomatic	7 (7.1%)	11 (8.1%)	1
Previous TIA	2 (2%)	7 (5.2%)	0.3
Previous Stroke	5 (5.1%)	4 (2.9%)	0.5

ICA, internal carotid artery; PSV, peak systolic velocity; EDV, end diastolic velocity;

*TIA, transient ischemic attack*.

Female patients who underwent CAS were older (76.4 ± 6.6 vs. 73.4 ± 8.2 years, *p* = 0.002), more likely to have hypertension (66.2 vs. 50%, *p* = 0.01), to be an active smoker (36.8 vs. 20.4%, *p* = 0.009) or a former smoker (19.1 vs. 7.1%, *p* = 0.01). In gynecological history, CAS patients were more likely to present menopause later with respect to CEA patients (49.5 ± 3.9 vs. 47.1 ± 4.3, *p* = 0.0001).

Technical procedural features for CEA and CAS are listed in Table 3.

**Table 3:** Procedural, technical features.

**Carotid Artery Endarterectomy Technical features**	
Anesthesia (*N*, %)	
General	18 (18.7%)
Loco-regional blockage	79 (80.6%)
Patch angioplasty (*N*, %)	88 (89.8%)
Eversion technique (*N*, %)	9 (9.2%)
Use of arterial shunt (*N*, %)	17 (17.3%)
Duration of the procedure (mean ± SD)	97 ± 23.7
**Carotid Artery Stenting Technical Features**	
Femoral Approach (*N*, %)	133 (97.8%)
Distal Cerebral Protection (*N*, %)	133 (97.8%)
Proximal Cerebral Occlusion (*N*, %)	3 (2.2%)
Aortic Arch Type (*N*, %)	
Type I	66 (48.5%)
Type II	52 (38.2%)
Type III	12 (8.8%)
Bovine	6 (4.4%)
Pre-dilatation (*N*, %)	4 (2.9%)
Stent design (*N*, %)	
Open Cell Stent	5 (3.6%)
Closed Cell Stent	112 (82.4%)
Double-layer Stent	19 (14%)
Post-dilatation (*N*, %)	128 (94.1%)
Access hemostasis (*N*, %)	
Arterial Closure device	90 (66.1%)
Manual Compression	46 (33.9%)
Procedure Duration (mean ± SD)	47.3 ± 20.2
Contrast media Used (mean ± SD)	21.4 ± 5.6

The vast majority of CEA procedures were performed under local, regional blockade (93.9%). Thirteen regional anesthesias were converted into general anesthesia during the operation. Patch angioplasty was the preferred surgical approach (89.8%), and an arterial shunt was used in 17.3%.

CAS procedures were conducted *via* a femoral approach in 97.8% of cases. Cerebral protection devices were used in 100%. In 97.8% of cases, a distal cerebral protection device was chosen.

Pre-dilatation was performed only in four cases (2.9%). The majority of stents implanted were characterized by a closed-cell design (*n* = 112; 82.4%); open-cell stents were used in only five cases (3.6%), and in 19 patients (14%), new double-layer stents were chosen. In 66.1% (*n* = 90), an arterial closure device was implanted at the end of the procedure. The average contrast medium used was 21.4 ± 5.6 cc.

A significant difference in duration of hospitalization was registered with a more extended stay for CEA patients (4.2 ± 1.8 vs. 3.3 ± 1.5 days, *p* = 0.0001).

### Early Outcomes

Acute technical success rates were 100% in CEA and 98.6% in CAS. Two endovascular procedures were interrupted due to the inability to engage the supra-aortic vessel after 20 min of the procedure (*n* = 1) or cross the culprit lesion (*n* = 1). Both cases were rescheduled, and CEA was performed in the following 24 h.

Minor early adverse events were 10 (10.2%) and 6 (4.4%) for CEA and CAS groups, respectively (*p* = ns). In detail: in the CEA group, five (5.1%) cervical hematomas were registered, determining the prompt surgical evacuation with no relevant consequences, and five patients suffered from cervical/cranial nerve injury that resolved in the following 6 months; in the CAS group, four (2.9%) post-CAS percutaneous access complications were recorded, including a pseudoaneurysm treated percutaneously with the thrombin injection and three groin hematomas that needed a surgical revision; one case of cerebral reperfusion syndrome occurred in the CAS group, mimicking a cerebral ischemic event that was clinically resolved in a few days.

At 30 days, any stroke and death rates were 2.9% for CAS and 0% for CEA.

One death occurred in the CAS group a few hours after the procedure due to cardiac complications.

Three TIAs (2.2%) were registered in the CAS group, which happened in the first 24 h after the procedure with complete neurological recovery before discharge. No strokes were recorded in the CAS group. In the CEA group, no TIA or strokes occurred at 30-day follow-up.

Subgroup analysis between symptomatic and asymptomatic carotid stenosis revealed that early ipsilateral TIA/stroke and cerebral reperfusion syndrome occurred in the asymptomatic group (TIAs *n* = 3; cerebral reperfusion syndrome *n* = 1), whereas the only post-procedural death happened in symptomatic carotid stenosis group (no procedure-related) with no statistical difference outlined (*p* = ns).

A complete revision of all early outcomes is shown in Table 4.

**Table 4:** Early in-hospital and 30-day outcomes.

	**CEA Group**	**CAS Group**	***p*-Value**
Duration of Hospitalization (mean ± SD)	4.2 ± 1.8	3.3 ± 1.5	0.0001
Cervical Hematoma (*N*, %)	5 (5.1%)	N/A	N/A
Percutaneous Access site complications (*N*, %)	N/A	4 (2.9%)	N/A
TIA (*N*, %)	0 (0%)	3 (2.2%)	0.26
Stroke (*N*, %)	0 (0%)	0 (0%)	
Death (*N*, %)	0 (0%)	1 (0.8%)	1
AMI (*N*, %)	0 (0%)	0 (0%)	
Renal Impairment (*N*, %)	0 (0%)	0 (0%)	
Cerebral Reperfusion Syndrome (*N*, %)	0 (0%)	1 (0.8%)	1

*TIA, transient ischemic attack; AMI, acute myocardial infarction*.

### Late Outcomes

The mean follow-up values were 33.8 ± 22.9 and 35.8 ± 24.4 months in the CAS and CEA groups, respectively. Twenty-six patients died during the study period: 9 in the CEA group, and 17 in the CAS group. No deaths were related to ipsilateral carotid cerebral ischemic/hemorrhagic events. In the CEA group, three deaths were related to cardiac complications; two deaths occurred due to intracranial hemorrhages after a trauma; the remaining four occurred during hospitalizations for general age-related clinical worsening in the oncological setting. In the CAS group, eight deaths were related to cardiac pathologies, five were related to cancer, and the remaining were related to age-related general decay.

**Figure 1:**
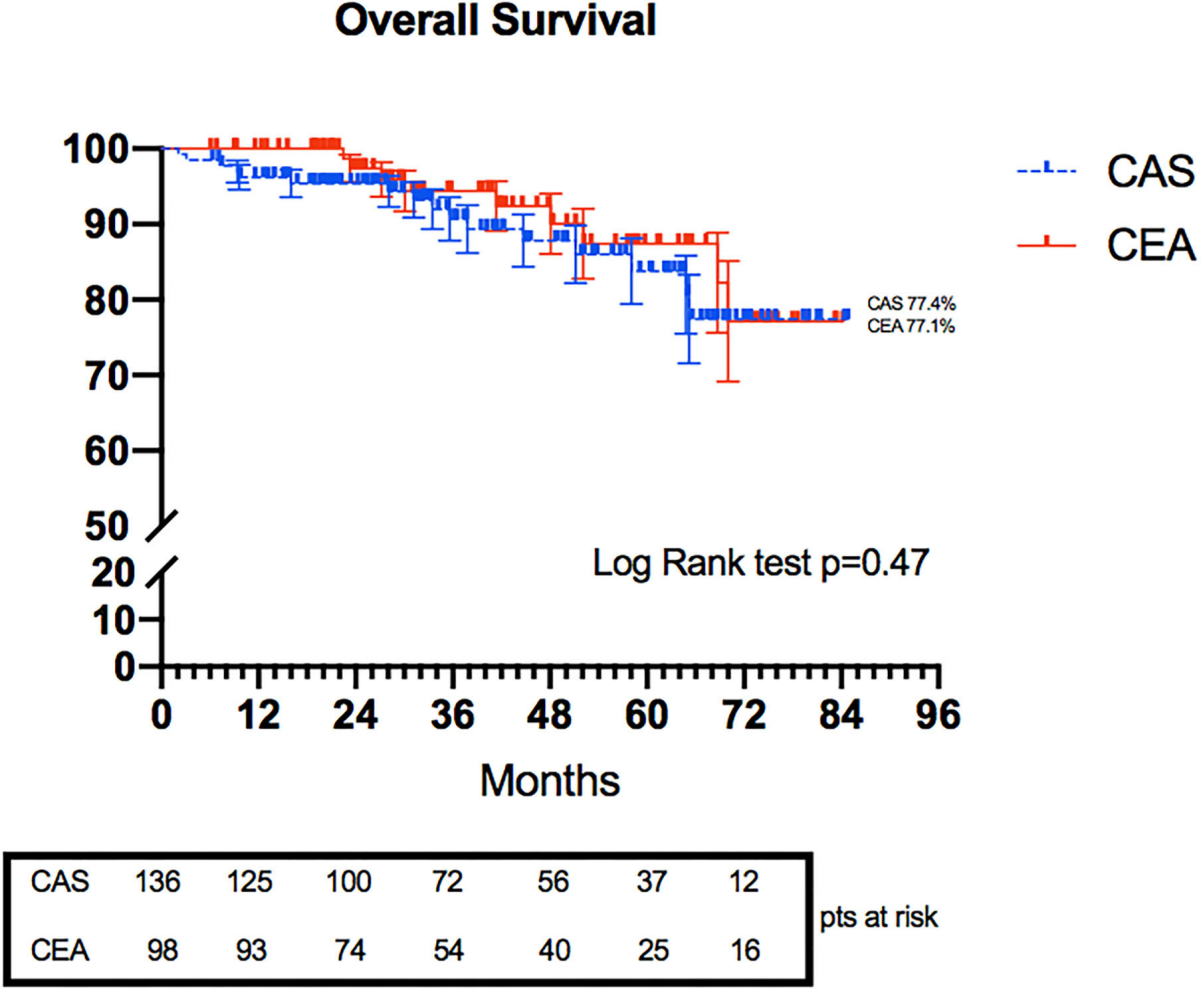
Kaplan–Meier curves representing overall survival rates in CAS and CEA groups.

Kaplan–Meier overall survival rate highlighted no statistical differences at 6 years (77.4 vs. 77.1% in CAS and CEA groups, respectively, *p* = 0.47) (Figure 1).

During the observational time, eight ipsilateral ischemic events were registered: five in the CAS group (one major stroke and three TIAs) and three TIAs in the CEA group.

**Figure 2:**
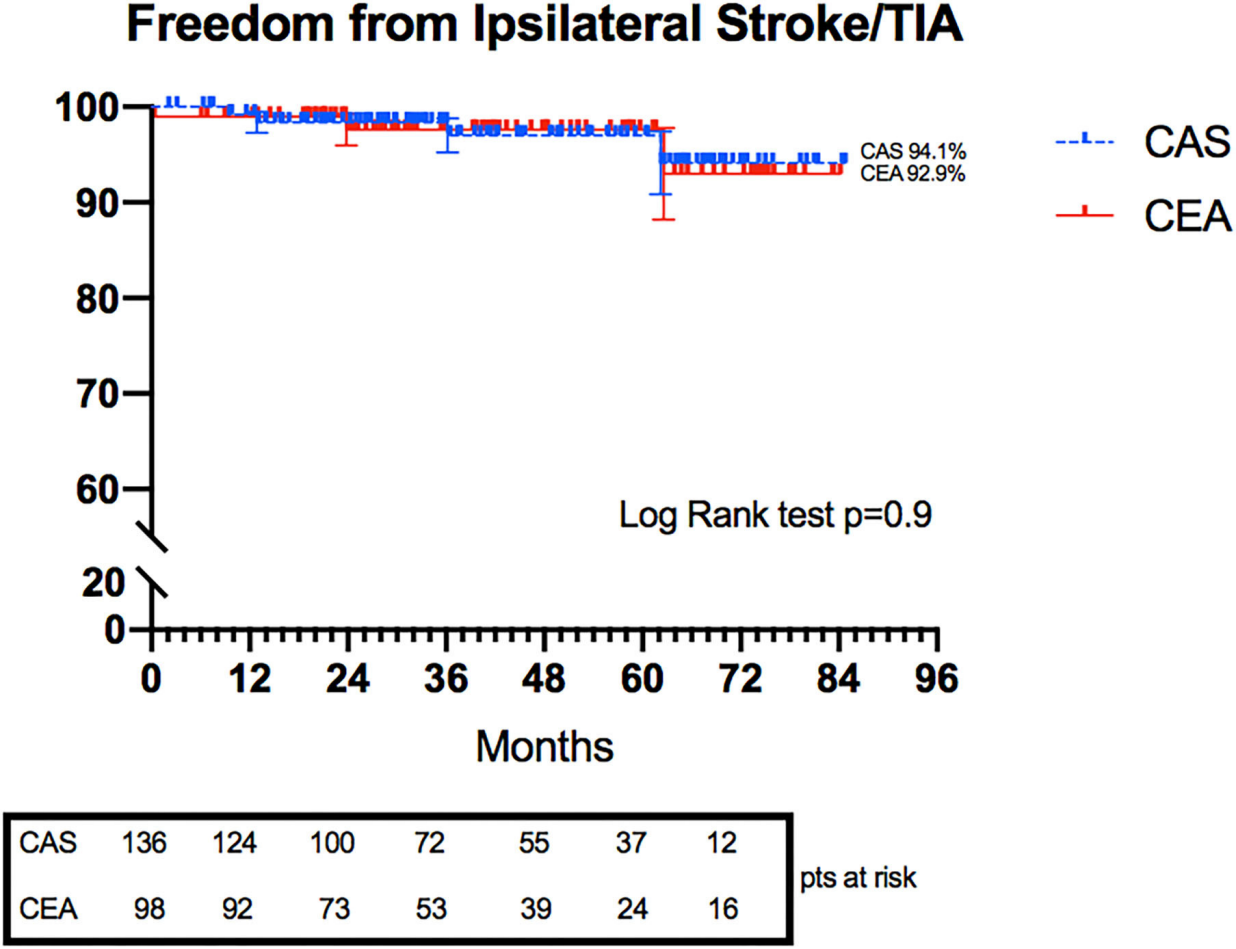
Kaplan–Meier curves representing freedom from Ipsilateral Stroke/TIA in CAS and CEA groups.

Freedom from ipsilateral stroke/TIA was also evaluated: no statistical difference was shown between the CAS and CEA groups at 6 years (94.1 vs. 92.9%, *p* = 0.9) (Figure 2).

**Figure 3:**
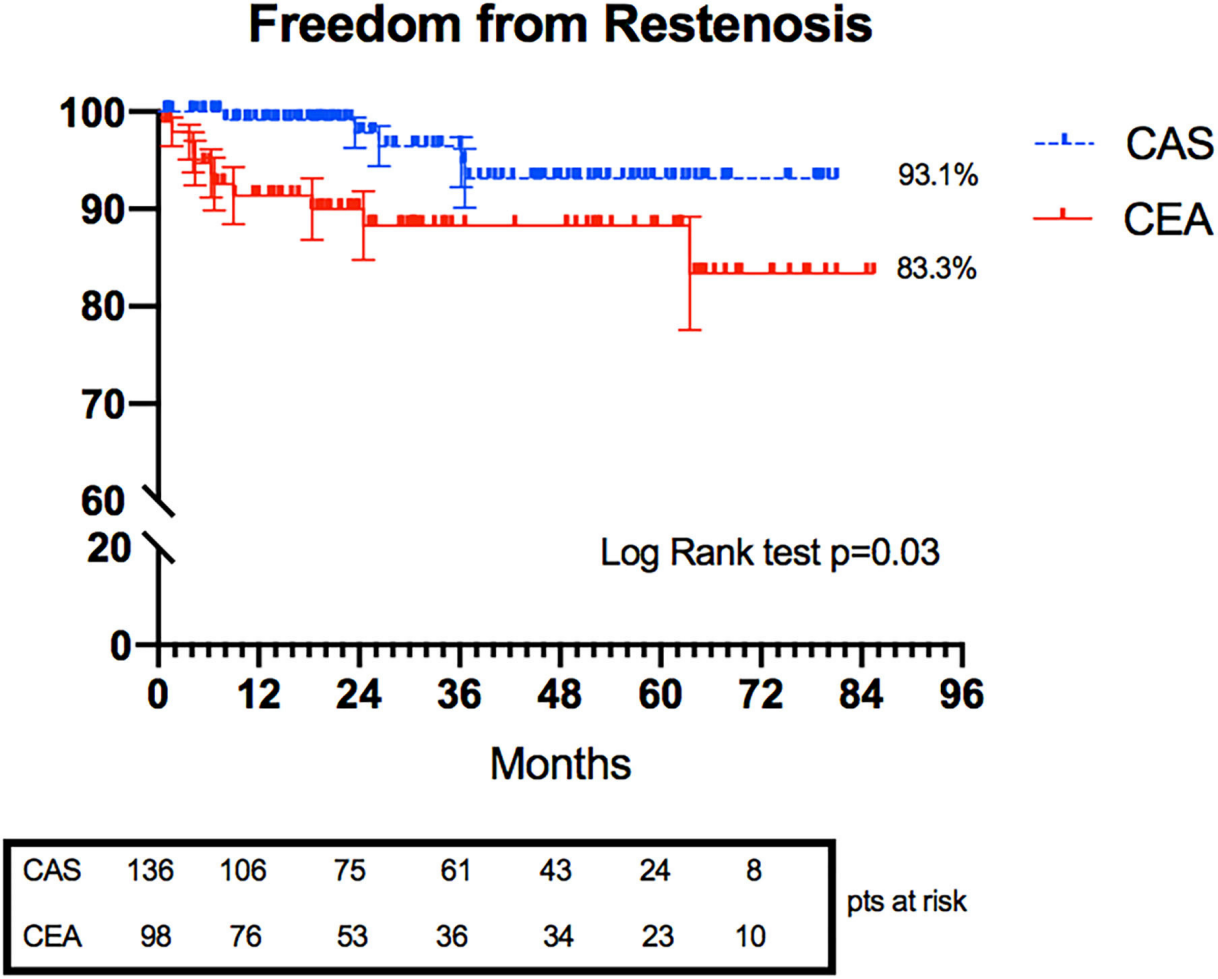
Kaplan–Meier curves representing freedom from restenosis in CAS and CEA groups.

The rates of treated carotid restenosis at 6 years were 93.1 and 83.3% in CAS and CEA, respectively (*p* = 0.03) (Figure 3).

Three restenoses occurred in the CAS group, with symptomatic stent thrombosis in one case. In the CEA group, nine restenoses were registered; two were symptomatic, leading to TIA manifestation.

**Figure 4:**
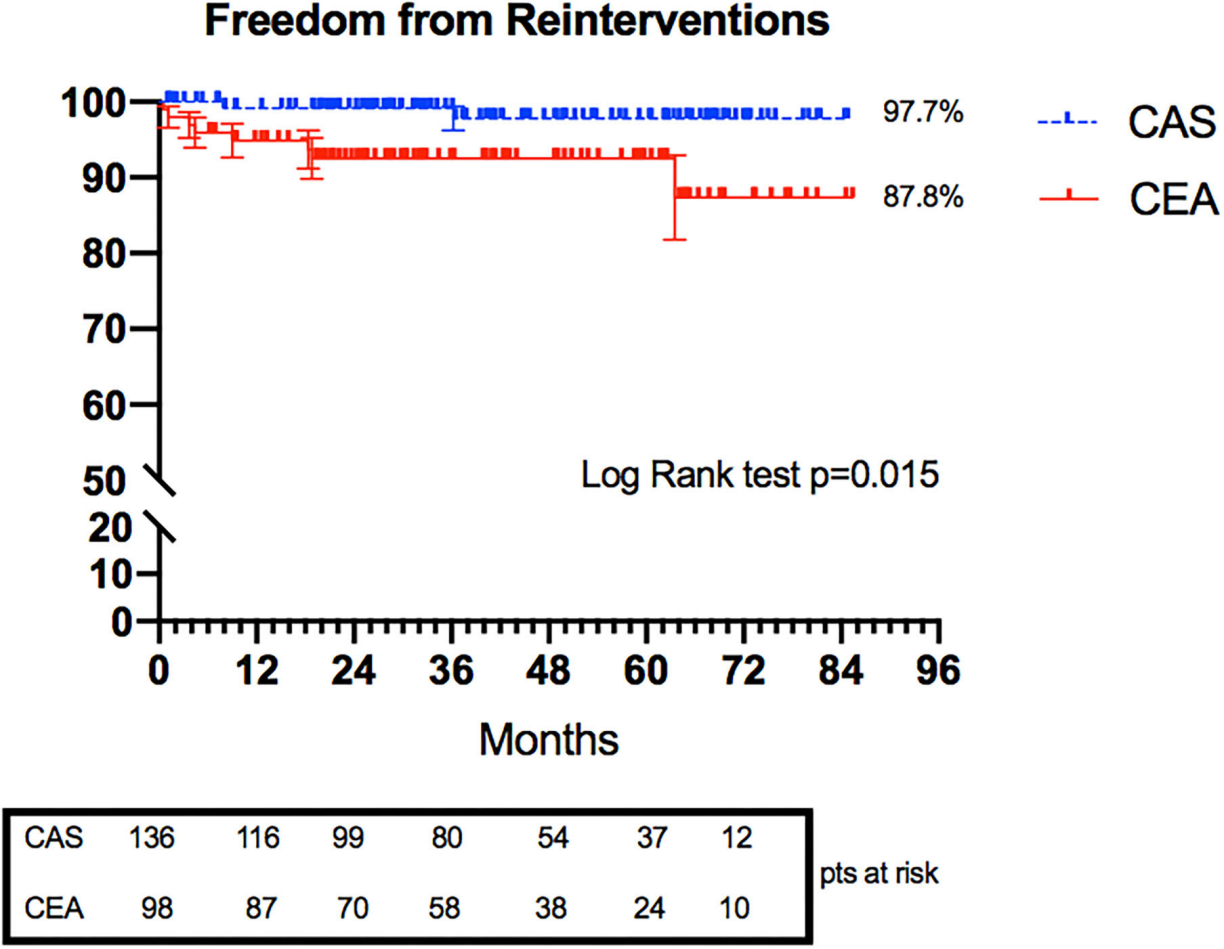
Kaplan–Meier curves representing freedom from reinterventions in CAS and CEA groups.

Freedom rates from reintervention at 6 years were 97.7 and 87.8% in the CAS and CEA groups, respectively (Figure 4) (*p* = 0.015).

Eleven reinterventions were registered: eight in the CEA group and three in the CAS group. All reinterventions in the CEA group were related to >50% restenosis with hemodynamic impairment of the treated carotid. In the CAS group, one reintervention was for stent thrombosis and included endovascular thromboaspiration and re-stenting, one reintervention was for early proximal stent migration and consisted of new stent implantation distally, and one reintervention was for severe restenosis due to stent recoil treated by re-stenting and angioplasty.

## Discussion

Large vessel cerebrovascular disease is linked to 15–20% of ischemic stroke, and internal carotid artery stenosis represents the cause of more than half of these events (23, 24).

Facing vascular diseases in women represents a sensitive point for vascular surgeons, majorly related to anatomic and pathophysiologic differences: considerably small carotid diameter, higher arterial velocity in the carotid artery, different distribution of atherosclerotic plaque, and different arterial wall compliance with higher age-related stiffness with a consecutive higher risk of thrombotic/embolic events (8).

In this scenario, carotid artery revascularization procedures in women need focused planning, according to anatomy, physiology, and vascular surgeons' experience. Moreover, women who undergo carotid procedures are usually older, are more often obese, more often have hyperlipidemia, hypertension, and are later referred to a vascular specialist, leading to an underuse of medical therapy and delay in surgical indication and treatment (25–27).

Large randomized clinical trials (RCTs) regarding female carotid revascularization benefits are underpowered because of the under-representation of the women population with respect to male counterparts. Besides, most of these trials were performed nearly two decades ago, when medical treatment and operative techniques were quite different from the new possibilities.

The number of women enrolled in the North American Symptomatic Carotid Endarterectomy Trial, the Asymptomatic Carotid Atherosclerosis Study (ACAS), and the European Carotid Surgery Trial (28–30) was limited, varying from 28 to 34%. Sex subgroup analysis revealed that CEA might not be as useful in women as it is in men. The ACAS trial also showed that CEA reduced the 5-year rate of ipsilateral stroke or perioperative death only by 17% in asymptomatic women for 66% in asymptomatic men.

In this perspective, vascular specialists' behavior in treating women with the carotid disease has been cautious, reinforced by several “gray-zone” data far from experience presented in this report.

In perioperative and early outcomes, our study demonstrated low rates of perioperative ischemic cerebral events (2.2 vs. 0%, in CAS and CEA groups, respectively) and death (0.8 vs. 0%, in CAS and CEA groups, respectively) for both groups without significant statistical differences.

Nevertheless, the higher number of CAS group events could be related to a more complex medical condition of patients (age and comorbidities), resulting in a higher perioperative risk.

The early safety of both techniques could lead to a substantial reappraisal of the female sex role as an independent risk factor for this kind of approach. We believe that a careful patient selection and the operator's high experience may compensate for the influence that female sex could have on these procedure's outcomes, especially in the CAS approach in which the use of a standardized procedural protocol and risk stratification is essential to reduce the incidence of adverse events.

Data from RCTs showed results that differ substantially from our experience. In the Carotid Revascularization Endarterectomy vs. Stenting Trial (CREST), asymptomatic females treated with CEA had composite rates of death/stroke/MI and stroke at 30 days of 3.7 and 1.6%, respectively (31). Similar results were from the ACAS trial (composite rate of death/stroke of 3.6%) (28).

Comparing CAS with CEA procedures in women, RCTs revealed a preponderant higher risk of ipsilateral stroke/death in the CAS group. In the CREST, rates of periprocedural endpoints for combined symptomatic/asymptomatic female patients (stroke/death/MI) after CAS were 6.8%, significantly higher than CEA women (hazards ratio 1.84, *p* = 0.047). Periprocedural stroke alone in CAS was more than twofold than CEA, respectively, 5.5 vs. 2.2% (29).

Even higher rates resulted from the Stent-Protected Angioplasty vs. Carotid Endarterectomy trial in which periprocedural ipsilateral stroke/death in CAS symptomatic women reached 8.2 vs. 6% in the CEA counterpart. In female patients, the combined rate of ipsilateral stroke/death within 2 years, plus periprocedural stroke and death, was higher in the CAS group (8.3%) than the CEA group (6.7%) (32).

An important analysis of more than 20,000 women undergoing carotid revascularization found that CAS in symptomatic patients was related to a higher risk of perioperative morbidity and mortality compared with CEA (combined perioperative mortality/mortality was 10.9% for CAS vs. 3.8% for CEA), with a lower difference for asymptomatic patients (3.1% in CAS vs. 1.7% in CEA) (33).

Bisdas et al. (34) confirmed the same assumption for symptomatic women, but comparable outcomes were highlighted in asymptomatic female patients for both procedures.

The Stent-Protected Angioplasty vs. Carotid Endarterectomy, Endarterectomy vs. Angioplasty in Patients With Symptomatic Severe Carotid Stenosis, and the Stenting and Angioplasty With Protection in Patients at High Risk for Endarterectomy trials compared CAS with CEA in symptomatic patients: outcomes in female patients were quite weak in CAS risks. Minimal data were outlined from the Endarterectomy vs. Angioplasty in Patients With Symptomatic Severe Carotid Stenosis and the Stenting and Angioplasty With Protection in Patients at High Risk for Endarterectomy trials in which women were strongly underrepresented (35–37).

In restenosis, female sex has been widely recognized as an independent risk factor after carotid revascularization procedures (38). Kumar et al. (39) published a recent meta-analysis in which in evaluated restenosis rate after carotid interventions, no specific data about female patients were reported, but their results could help understand the real role of sex; in fact, they reported a rate of 5.8% for restenosis >70% or occlusion after any type of CEA over a mean follow-up of 47 months. Conversely, over a mean follow-up of 60 months, the rate of restenosis >70% or occlusion after CAS or balloon angioplasty reached 10.3%.

The CREST trial showed a general risk of restenosis (>70%)/occlusion at 2 years of 6.0 and 6.3% in the CAS and CEA groups, respectively. Our data differ significantly from these numbers, especially for CAS procedures. Our study's patients are all women, and our data are referred to restenosis >50%, reinforcing the substantial discrepancy highlighted.

De Rango et al. (40) compared CAS with CEA procedures in female patients, highlighting a non-inferiority of CAS with respect to CEA. Our results confirm the data presented in their paper; perioperative risks of stroke or death were no different whether they were symptomatic or not (1.9 vs. 3% in CAS and CEA, respectively). Late outcomes in survival and freedom from ipsilateral stroke and restenosis did not differ significantly; in fact, Kaplan–Meier curves revealed no differences between the CAS and CEA groups for any periprocedural stroke/death and ipsilateral stroke at 5 years (4.1 vs. 8.1%; *p* = 0.18). Moreover, 5-year rates of restenosis >50% were nonsignificantly higher in women after CEA than after CAS (1.8 vs. 8.1%; *p* = 0.058).

Our data in overall survival rate are worse than other studies (29). This consideration could be related to a higher average age of the enrolled patients at the procedure time. Restenosis and reintervention rate at 6 years was significantly higher in the CEA group, confirming a trend already highlighted in previous studies. Freedom from ipsilateral ischemic events at 6 years was similar between the two groups.

Literature offers different and conflicting views of the real impact that carotid revascularization (especially CAS) has on female patients. Clinical data from everyday experiences represent an essential source of evidence, especially in this specific topic in which clinical trials' outcomes are relatively weak.

## Limitations

The retrospective, nonrandomized nature of the study represents one of the significant limitations. Patients enrolled were mostly asymptomatic, and plaque characteristics, vascular anatomy, patient's general condition, and patient's preference were all elements used to decide whether CAS or CEA was appropriate, and regional anesthesia was routinely used for CEA. All these elements influenced the operator's decision to choose the best option for each patient and eventually to a possible selection bias that could have impacted the results.

Both procedures were made by experienced vascular surgeons able to perform both techniques in a high-volume vascular unit; in this perspective, the same outcomes in the female population could not be assured in other health-care settings.

Lastly, an essential difference in numerosity between symptomatic and asymptomatic patients has been registered; for this reason, no further analysis between these two groups has been made.

## Conclusion

Our results highlighted that carotid revascularization procedures in female patients were safe and effective in preventing future ischemic cerebral events. Carotid stenting represents an acceptable alternative to CEA in symptomatic and asymptomatic women when performed by experienced operators. Based on these findings, a future trial focused on women alone could be worthy of consideration to understand the role of these two procedures in future clinical practice to overcome the longstanding lack of representation of women in clinical trials.

## Data Availability Statement

The raw data supporting the conclusions of this article will be made available by the authors, without undue reservation.

## Author Contributions

EP, GD, CS, and GP: conception and design. EP, GD, GA, BB, and CP: analysis and interpretation. EP, GA, BB, and CP: data collection. EP, GD, GA, and BB: statistical analysis. EP, GD, GA, BB, and CP: writing the article. EP, GD, CS, AC, and GP: critical revision of the article. EP, GD, GA, BB, AC, CS, and GP: final approval of the article. EP: overall responsibility. All authors read and approved the final version of the manuscript.

## Conflict of Interest

The authors declare that the research was conducted in the absence of any commercial or financial relationships that could be construed as a potential conflict of interest.

## Abbreviations

CEA: carotid artery endarterectomy
CAS: carotid artery stenting
NIRS: near infrared spectroscopy
PSV: peak systolic velocity
EDV: end diastolic velocity
MI: myocardial infarction
MEAE: minor early adverse events
RCT: randomized clinical trial.
